# CD71^+^ erythroid suppressor cells impair adaptive immunity against *Bordetella pertussis*

**DOI:** 10.1038/s41598-017-07938-7

**Published:** 2017-08-10

**Authors:** Afshin Namdar, Petya Koleva, Shima Shahbaz, Stacy Strom, Volker Gerdts, Shokrollah Elahi

**Affiliations:** 1 Department of Dentistry, Faculty of Medicine and Dentistry, University of Alberta, Edmonton, T6G 2E1 Alberta Canada; 2 Department of Medical Microbiology and Immunology, Faculty of Medicine and Dentistry, University of Alberta, Edmonton, T6G 2E1 Alberta Canada; 3 Vaccine and Infectious Disease Organization, International Vaccine Centre, University of Saskatchewan, Saskatoon, S7N 53E SK Canada; 4 Department of Veterinary Microbiology, Western College of Veterinary Medicine, University of Saskatchewan, Saskatoon, S7N 5E3 SK Canada

## Abstract

Infant’s immune system cannot control infection or respond to vaccination as efficiently as older individuals, a phenomenon that has been attributed to immunological immaturity. Recently, we challenged this notion and proposed the presence of actively immunosuppressive and physiologically enriched CD71^+^ erythroid cells in neonates. Here we utilized *Bordetella pertussis*, a common neonatal respiratory tract pathogen, as a proof of concept to investigate the role of these cells in adaptive immunity. We observed that CD71^+^ cells have distinctive immunosuppressive properties and prevent recruitment of immune cells to the mucosal site of infection. CD71^+^ cells ablation unleashed induction of *B. pertussis*-specific protective cytokines (IL-17 and IFN-γ) in the lungs and spleen upon re-infection or vaccination. We also found that CD71^+^ cells suppress systemic and mucosal *B. pertussis*-specific antibody responses. Enhanced antigen-specific adaptive immunity following CD71^+^ cells depletion increased resistance of mice to *B. pertussis* infection. Furthermore, we found that human cord blood CD71^+^ cells also suppress T and B cell functions *in vitro*. Collectively, these data provide important insight into the role of CD71^+^ erythroid cells in adaptive immunity. We anticipate our results will spark renewed investigation in modulating the function of these cells to enhance host defense to infections in newborns.

## Introduction

Neonates are highly susceptible to disseminated and fetal infections^1^. The clear example of this is HIV infection, which rapidly progresses to AIDS in newborns in the absence of antiretroviral therapy^2, 3^. Similarly, herpes simplex virus (HSV) infection rarely causes severe disease in healthy individuals after a few months of life, but among neonates infected with HSV, mortality rates are as high as 85% without aggressive treatment^4^. Infant’s immune system cannot control infection or respond to vaccination as efficiently as older children or adults, a phenomenon that has been attributed to immunological immaturity^5^. While most vaccines do induce protective immunity in older children and adults, their efficacy in the very young often requires further manipulation and optimization. Concurrent with the development of ideas about the relevance of the type (i.e.Th1&Th2) of immune response, studies suggest that neonates are able to respond to antigen, but with a Th2-type bias^6^. On the other hand, newborns have limited immunological memory reflecting the fetal life, where exposure to antigens is highly restricted to non-inherited maternal alloantigens (NIMA)^7^. This lack of immunological memory increases their susceptibility to infectious diseases which accounts for 40% of the annual 3 million worldwide neonatal mortality^8^. Another important factor might be lower frequency of immune cells in neonates compared to adults^5^. Furthermore, qualitative and phenotypic differences in neonatal T cells, B cells and antigen presenting cells (APCs) are also reported, suggesting the neonatal adaptive and innate immune cells to be underdeveloped^5^. However, other reports demonstrate that neonates have competent immune system and under certain circumstances are able to mount T-cell responses comparable to adults *in vivo* ^9–11^. It has become progressively clear that neonatal adaptive immune responses exhibit a great deal of variability ranging from lack of response to fully functional^5^. This variability may explain presence of some immunological factors or non-immune cells in newborns that may impact their immune responses.

In agreement, we recently, described a possible mechanism that provides considerable insight into the reduced immunity in young infants. We found that erythroid precursor cells, co-expressing the transferrin receptor CD71 and erythroid marker TER119, are present in impressively high numbers in newborn mice and human cord blood (CD71^+^CD235a^+^)^12^. Their presence after birth contribute to the extreme vulnerability of neonates to severe infections, as ablation of CD71^+^ erythroid cells (CD71^+^ cells) or their gradual decline by age restores resistance to prenatal pathogens^12^. More importantly, we showed that the functionality of adoptively transferred adult CD11b^+^ granulocyte/macrophage and CD11c^+^ dendritic cells wiped out in the newborn mouse in response to *Listeria monocytogenes* infection, whereas the functionality of donor neonatal CD11b^+^CD11c^+^ cells into the adult mouse was restored^12^. This further demonstrates the immunosuppressive nature of the neonatal environment. In addition, we have recently demonstrated that CD71^+^ cells impair innate immune responses against *Bordetella pertussis* in newborn mice (accepted article﻿ i﻿n The Journal of Immunology).

Pertussis (whooping cough) is a highly contagious bacterial disease mainly caused by *B. pertussis* and occasionally by *B. parapertussis* ^13, 14^. Resurgence of pertussis has been reported in recent years worldwide^15, 16^ and sadly, the highest rate of complications and/or pertussis-related mortality are consistently observed in neonates who are too young to be vaccinated or who have yet to receive their primary immunization series^17–19^. Therefore, better understanding the nature of protective immunity could enable us in designing an effective approach in generating an earlier protective immune response against infectious diseases such as pertussis in the neonate.

In terms of immune correlates of protection against pertussis, it has been reported that antibodies play an essential role in bacterial toxin neutralization and in the prevention of bacterial attachment to the host cells^20, 21^. More importantly, vaccine-induced antibodies against *B. pertussis* virulence factors such as pertussis toxin (Ptx), fimbria (fim 2 and fim 3) and pertactin are shown to be protective^22–26^. In addition to antibodies, CD4^+^ T cells and Th1-like cytokines are shown to play a protective role against *B. pertussis* ^27, 28^. T cell responses in immunized children, as well as animal models following immunization with the whole vaccine (Pw) are a Th1 type immune response^29^, however, immunization with acellular pertussis vaccine (Pa), induces a Th2-baised or mixed Th1/Th2 immune profile^24, 30^. Several studies have shown that IFN-γ plays a central role in both innate and adaptive immunity against *B. pertussis*, since IFN-γ ^−/−^ or INF-γ defective mice develop lethal infection following intranasal challenge^31, 32^. More recent evidence demonstrated that both Th1 and Th17 T cells contribute to the clearance of *B. pertussis*, and that IFN-γ has an instrumental role in adaptive immunity to bacterial clearance^33^. Here we test the hypothesis that physiologically enriched CD71^+^ cells in neonates suppress their adaptive immune responses to pathogens. Pertussis has been used as a proof of concept model to investigate whether and how CD71^+^ cells impact adaptive immune responses in this model. Our results here for the first time demonstrate that CD71^+^ cells in addition to innate immunity also suppress adaptive immunity in the newborn.

## Results

### Depletion of CD71^+^ cells increased accumulation of immune cells in the lungs of newborn mice following challenge with *B. pertussis*

**Figure 1:**
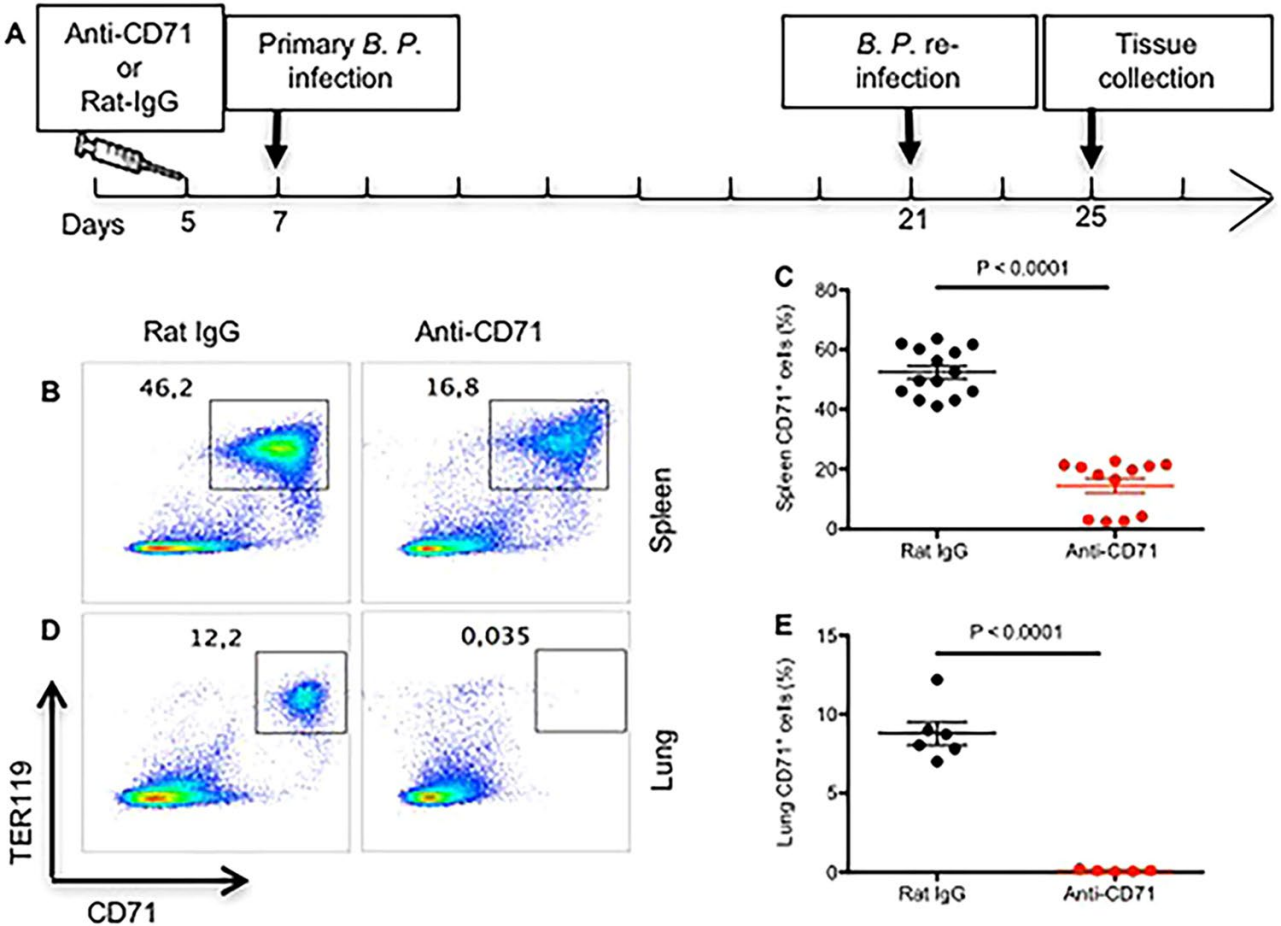
Anti-CD71 antibody significantly depletes CD71^+^ erythroid cell in the lungs and spleen on newborn mice. (**A**) The cartoon shows intervention time points. (**B,D**) Representative plots showing percent CD71^+^Ter119^+^ in the spleen and lungs for isotype (Rat-IgG) treated compared with anti-CD71 treated mouse. (**C–E**) Percent CD71^+^ cells in the spleen and lungs for anti-CD71 treated versus controls, day 2 post treatment.

We have previously shown that CD71^+^ cells impair innate immune responses against neonatal pathogens^12^. In addition, we have demonstrated that CD71^+^ cells hinder innate immune responses to *B. pertussis* (acc﻿epted article in The ﻿Journal of Immunology). In this regard, we initially re-assessed the frequency of CD71^+^TER119^+^ cells after treatment with anti-CD71 antibody. Five-day old newborn mice were either treated with anti-CD71 antibody (200 μg) or Rat IgG isotype using i.p. injection and the proportion of CD71^+^TER119^+^ cells 2 days after treatment was evaluated by flow cytometry. As we expected, anti-CD71 antibody significantly reduced percentages of CD71^+^TER119^+^ cells in the spleen and lungs of newborn mice (P < 0.0001; Fig. 1B,C) and (P < 0.0001; Fig. 1D,E), respectively.

**Figure 2:**
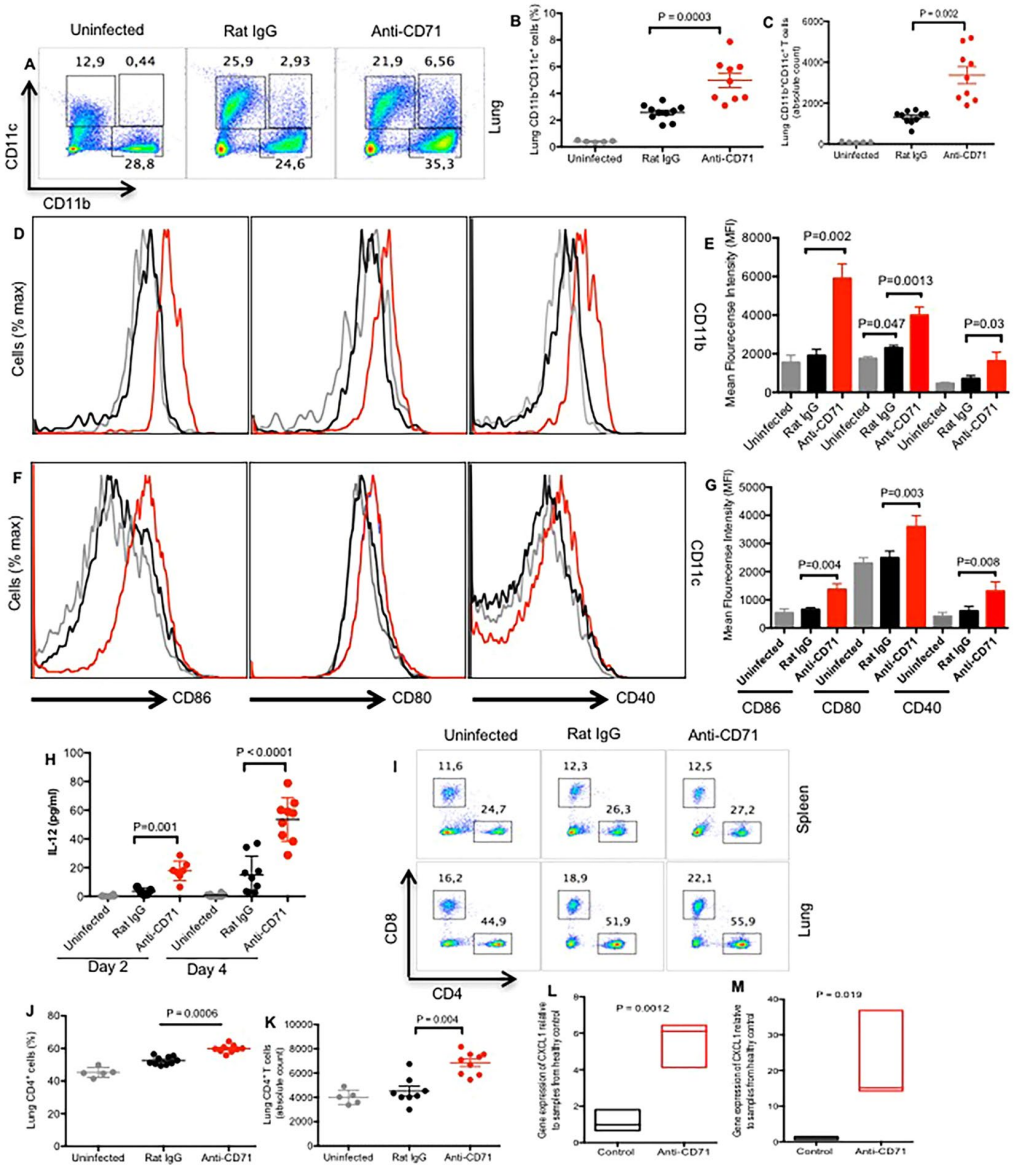
Deletion of CD71^+^ cells unleashes recruitment and function of immune cells in the lung in response to *B. pertussis* low dose infection. (**A**) Representative dot plots showing percentages of CD11b^+^, CD11c^+^ and CD11b^+^CD11c^+^ cells in the lungs of newborns day 2 post infection with *B. pertussis*. (**B,C**) Percentages and absolute number of CD11b^+^ and CD11b^+^CD11c^+^ cells isolated from the digested lung tissues obtained from the anti-CD71 treated versus isotype control at day 2 post infection. (**D–G**) Relative CD86, CD80, and CD40 expression by CD11b^+^ or CD11c^+^ cells recovered from the lungs at day 2 post infection, treated with anti-CD71 (red histograms gated on CD11b^+^ or CD11c^+^cells) or isotype control antibody (black histograms gated on CD11b^+^or CD11c^+^ cells) and uninfected mice (gray histograms gated on CD11b^+^or CD11c^+^ cells) administered 200 μg of each antibody at day 5. (**H**) IL-12 levels recovered from the lung homogenate of mice at day 2 and 4 post infection, treated with anti-CD71, isotype control antibody or uninfected mice. (**I**) Representative plots showing percentages of CD4^+^ and CD8^+^ T cells. (**J**,**K**) Percentages of CD4^+^ and CD8^+^ T cells from the digested lung tissues at day 2 post infection, either treated with anti-CD71 or isotype control compared with each other and uninfected mice. (**L**,**M**) CXCL-1 and CXCL-2 genes expression in the lungs of anti-CD71 or isotype control treated mice after *B. pertussis* infection compared with uninfected mice. Each point represents data from an individual mouse, representative of at least three independent experiments. Bar, mean ± one standard error.

Recently, we have shown that depletion of CD71^+^ cells does not impact immune cells recruitment or activation into the lungs or spleen in the absence of infection^12^. Here we investigated infiltration of immune cells into the lungs and spleen of newborn mice either treated with anti-CD71 antibody or Rat IgG isotype control compared to uninfected controls at day 5 of age and challenged intranasally with *B. pertussis* (~5 × 10^2^ CFUs) 48 hours later. The spleens and lungs of neonates were harvested at day 2 post-infection and subjected to immune phenotyping. As indicated in Fig. 2A–C, depletion of CD71^+^ cells resulted in significant infiltration of CD11b^+^CD11c^+^ and CD11b^+^ cells into the lungs of newborns. Importantly, we observed that lung CD11b^+^ and CD11c^+^ cells from CD71^+^ cell depleted neonatal mice significantly upregulated expression of costimulatory molecules CD40, CD80, and CD86 compared to isotype treated controls (Fig. 2D–G). However, this was not the case for the spleen CD11b^+^ and CD11c^+^ (data not shown). Interestingly, we observed significantly higher levels of IL-12 in the lungs of CD71^+^ cells depleted mice (Fig. 2H). Similarly, the percentage and absolute number of CD4^+^ T cells infiltrated into the lungs of CD71 treated neonates were also increased (P = 0.0006 and P = 0.004 respectively; Fig. 2I–K), but this was not the case for CD8^+^ T cells (P = 0.1; data not shown). We further examined the gene expression of pro-inflammatory chemokines (CXCL1, CXCL2 and CCL2), chemokine receptor CCR7, and TLR4 in lung tissues in order to determine the potential mechanism(s) of immune cells infiltration into the lungs of newborns following low dose infection with *B. pertussis*. We found a significant increase in the gene expression of CXCL1 (P = 0.0012) and CXCL2 (P = 0.019) chemokines in anti-CD71 treated versus isotype control treated mice when compared with uninfected group (Fig. 2L,M); however, no significant difference in the gene expression of CCL2, CCR7 and TLR4 was observed between the groups (data not shown).

### Depletion of CD71^+^ cells enhanced *B. pertussis-*specific T cell response

**Figure 3:**
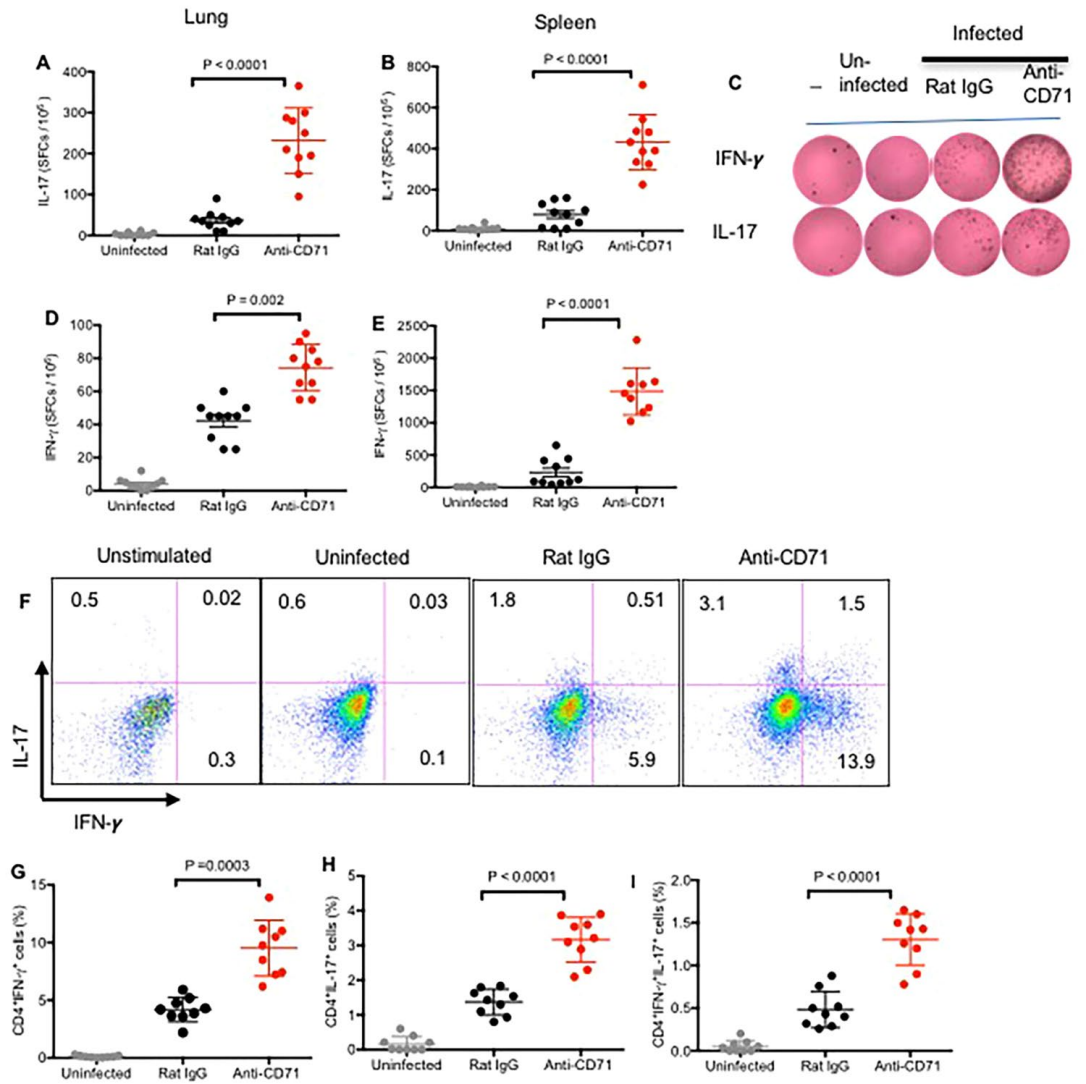
Depletion of CD71^+^ cells enhances IL-17 and IFN-γ secretion by adaptive immune cells. **(A**) Lung cells or (**B**) splenocytes (5 × 10^4^) from anti-CD71 treated or control mice were stimulated with medium alone or with HKBP for 24 h, IL-17 secreting cells were visualized and enumerated by ELISpot. (**C**) Representative ELISpot images are shown. (**D**) Lung cells or (**E**) splenocytes (5 × 10^4^) from anti-CD71 treated or control mice were stimulated with medium alone or with HKBP for 24 h, IFN-γ secreting cells were visualized and enumerated by ELISpot. (**F**) Representative plots showing IL-17 and IFN-γ producing CD4^+^ T cells among splenocytes following stimulation with HKBP for 6 h. (**G**) Percent CD4^+^ T cells secreting IFN-γ, H, secreting IL-17 and (**I**) secreting IFN-γ and IL-17 following stimulation with HKBP for 6 h. Each point represents data from an individual mouse, representative of three independent experiments. Bar, mean ± one standard error.

To determine whether depletion of CD71^+^ cells prior to primary infection enhances the mucosal and peripheral adaptive T cell response following re-infection, newborn mice were infected with high dose challenge (5 × 10^6^ CFUs). At day 4 post re-infection production of protective cytokines IL-17 and IFN-ϒ was assessed using ELISpot assay following re-stimulation of lung cells or splenocytes with HKBP (2 μg/ml) *in vitro*. We found that depletion of CD71^+^ cells prior to primary challenge with *B. pertussis* enhanced IL-17 production by the lung cells (P < 0.0001) as well as splenocytes (P < 0.0001) of mice (Fig. 3A–C). Similarly, depletion of CD71^+^ cells increased the production of IFN-ϒ by the lung cells (P = 0.002; Fig. 3C,D) and splenocytes (P < 0.0001; Fig. 3E) following stimulation *in vitro*. We further conducted intra-cellular cytokine assays to differentiate whether *B. pertussis* LPS is responsible for the induction of IFN-ϒ by innate immune cells or antigen-specific T cells are producing IFN-ϒ and IL-17. As shown in Fig. 3F–I, depletion of CD71^+^ cells enhanced IL-17 and IFN-ϒ secretion by CD4^+^ T cells following re-stimulation with HKBP *in vitro*. Although, CD4^+^ T cells from Rat-IgG isotype treated mice were capable of producing IFN-ϒ and IL-17 following re-stimulation, the magnitude of response was significantly lower compared with anti-CD71 treated group (Fig. 3G–I; P = 0.0003 and P < 0.0001 respectively).

### CD71^+^ cells impair induction of specific antibodies against *B. pertussis*

**Figure 4:**
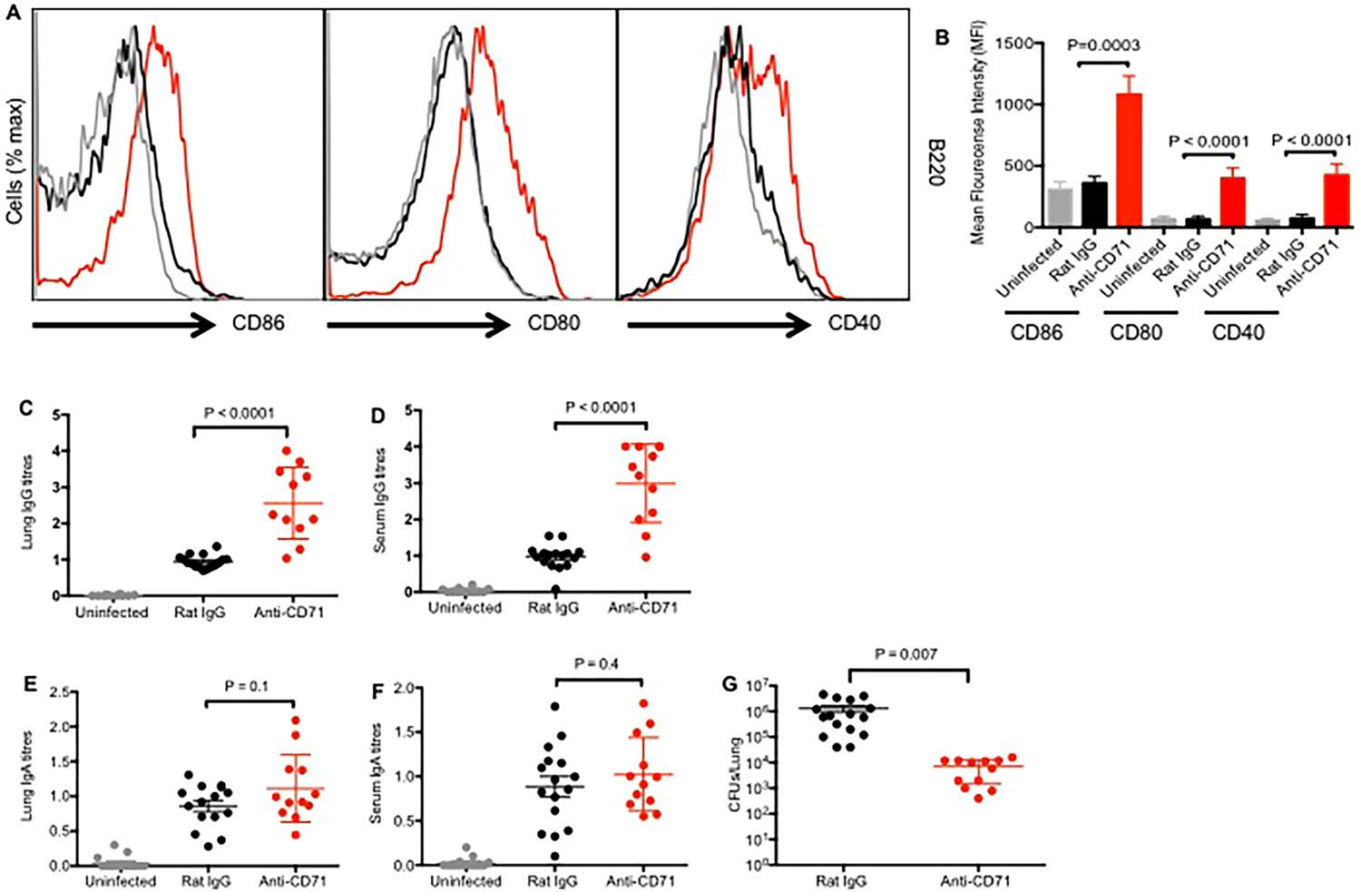
CD71^+^ cells inhibit *B. pertussis*-specific antibody response. (**A**) Relative CD86, CD80, and CD40 expression by B220 cells from the lungs at day 2 post infection, treated with anti-CD71 (red histograms gated on CD220 cells) or isotype control antibody (black histograms gated on CD220 cells) and uninfected mice (gray histograms gated on CD220 cells. (**B**) The mean fluorescent intensity for each parameter among the spleen CD220 cells from CD71 depleted mice was compared with isotype antibody treated or uninfected mice. (**C,D**) Anti-*B. pertussis*-specific IgG antibody detected in the lungs and serum respectively, (**E**,**F**) anti-*B. pertussis*-specific IgA antibody in the lungs and serum respectively were measured by ELISA as described in methods. (**G**) Recoverable bacteria in the lungs of mice after infection with *B. pertussis* (5 × 10^6^ CFUs) in control versus CD71^+^ cells depleted mice after re-infection. Each point represents data from an individual mouse, representative of three independent experiments. Bar, mean ± one standard error.

To determine the potential suppressive effects of CD71^+^ cells on humoral immunity, we evaluated the activation status of B cells post low dose *B. pertussis* challenge. Interestingly, we found B cells (B220 cells) become more activated when CD71^+^ erythroid cell were deleted by significantly upregulating expression of co-stimulatory molecules such as CD40, CD80 and CD86 compared to isotype treated and uninfected controls (Fig. 4A,B). Further to determine whether activation status of B cells following primary infection can impact humoral adaptive immune responses against *B. pertussis* infection, the levels of total IgG and IgA antibodies in serum as well as lung homogenates collected from mice 4 days post re-infection were measured. We observed that depletion of CD71^+^ cells prior to the low dose infection resulted in enhanced pertussis-specific IgG antibody in both the lung homogenates and serum of mice following re-infection (Fig. 4C,D). Interestingly, despite detectable levels of pertussis-specific IgA antibody in the lungs and serum of mice compared with non-vaccinated group, no significant differences were observed between anti-CD71 treated and control mice (Fig. 4E,F).

### Enhanced adaptive immune responses following depletion of CD71^+^ cells protected the mice against *B. pertussis* infection

Since depletion of CD71^+^ cells prior to primary infection with *B. pertussis* enhanced production of protective cytokines (e.g. IFN-γ and IL-17) and antibody response, we examined whether these immune responses would provide protection against *B. pertussis* infection. Therefore, mice were re-infected with *B. pertussis* (5 × 10^6^ CFU) two weeks post primary infection. Bacterial load in the lungs of mice was determined 4 days after re-infection. As depicted in Fig. 4G, enhanced adaptive immune responses against *B. pertussis* led to a significant reduction of bacterial load in the lungs of anti-CD71 treated mice compared to Rat IgG treated controls (P = 0.007).

### Depletion of CD71^+^ cells prior to immunization enhanced IL-17 and IFN-γ secreting CD4^+^ T cells against *B. pertussis*

**Figure 5:**
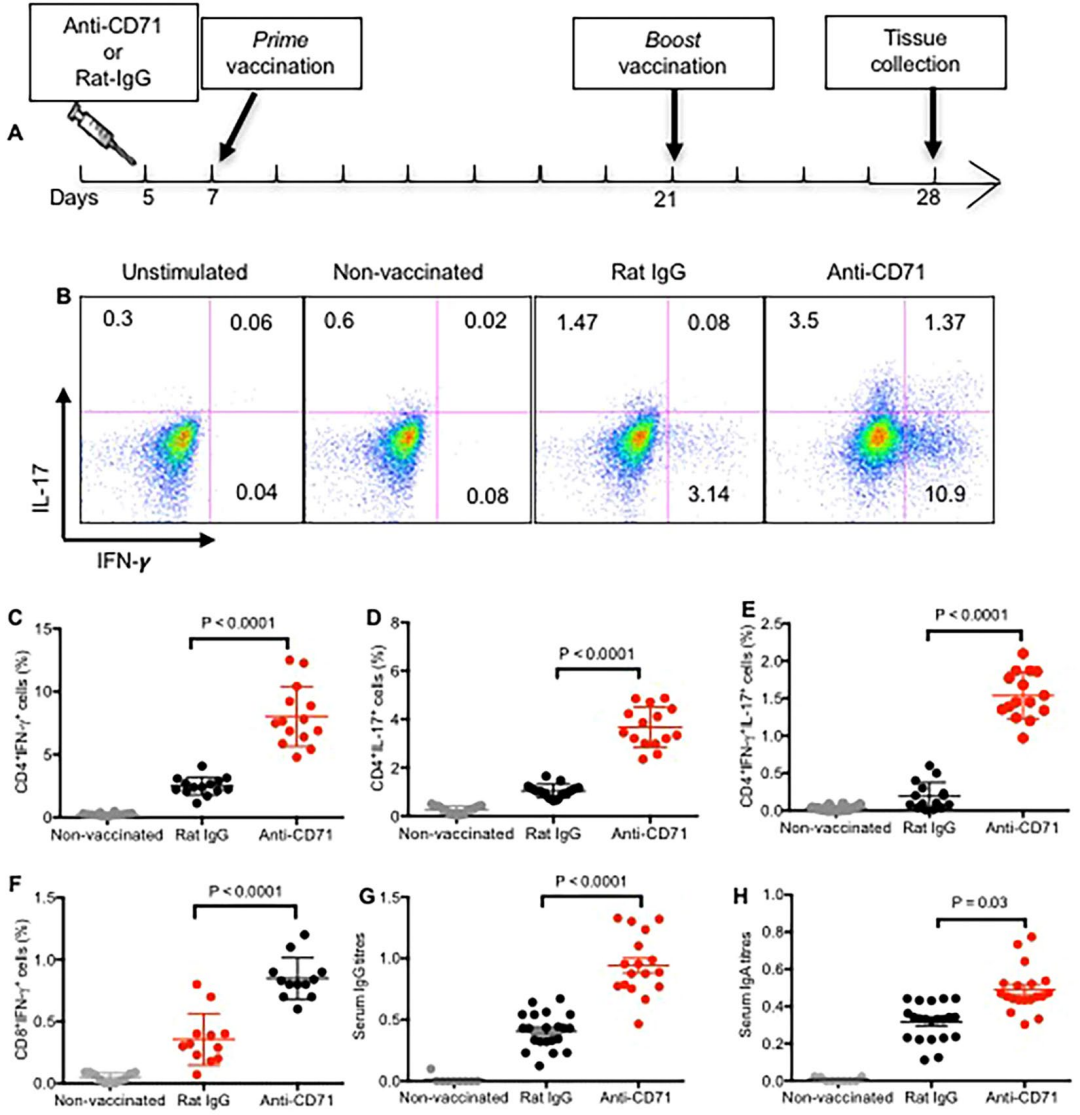
Depletion of CD71^+^ cells enhances IL-17, IFN-γ secreting and antibody producing adaptive immune cells following immunization. (**A**) The cartoon shows treatment and vaccination time points. (**B**) Representative plots showing IL-17 and IFN-γ producing CD4^+^ T cells among splenocytes following stimulation with HKBP for 6 h. (**C**) Percent CD4^+^ T cells secreting IFN-γ; (**D**) secreting IL-17 and (**E**) secreting IFN-γ and IL-17 following stimulation with HKBP for 6 h. (**F**) Percent CD8^+^ T cells secreting IFN-γ among splenocytes following stimulation with HKBP for 6 h. (**G**) Anti-*B. pertussis*-specific IgG antibody in serum, (**H**) anti-*B. pertussis*-specific IgA antibody in serum of either treated mice with anti-CD71 or isotype control group were measured by ELISA as described in methods. Each point represents data from an individual mouse, representative of three independent experiments. Bar, mean ± one standard error.

Immunization was performed to reconfirm our observations on the suppressive role of CD71^+^ cells on adaptive immunity following re-infection studies. Therefore, immunization of newborn mice with the adjuvant platform combination was performed as we have reported elsewhere^34, 35^. Newborn mice were either treated with anti-CD71 antibody or Rat IgG isotype at age of 5 day. They got vaccinated at day 7 and boosted two weeks later with the same vaccine as shown in Fig. 5A. Mice were euthanized one week post boost, splenocytes were stimulated with HKBP (2 μg/ml) and tested for the induction of IL-17 and IFN-γ by intracellular staining. Significant levels of IL-17 and IFN-γ were detected in CD4^+^ T cells following stimulation with HKBP *in vitro* from CD71^+^ cells depleted and vaccinated mice compared to non-vaccinated controls (Fig. 5B–E). Interestingly, we also observed very small proportion but significantly higher percentages of *B. pertussis*-specific CD8^+^ T cells in CD71 treated mice compared to vaccinated but treated with Rat IgG group (Fig. 5F).

### Depletion of CD71^+^ cells prior to immunization enhanced production of *B. pertussis*-specific antibodies

One week post boost, serum of mice were collected and tested for the presence of *B. pertussis*-specific antibodies (IgA & IgG). Significant levels of *B. pertussis*-specific antibodies, of both IgG- and IgA-isotypes, were detected from CD71^+^ cells depleted and vaccinated mice compared to Rat IgG treated and vaccinated group (Fig. 5G,H).

### Depletion of CD71^+^ cells prior to immunization protected the mice against infection with *B. pertussis*

**Figure 6:**
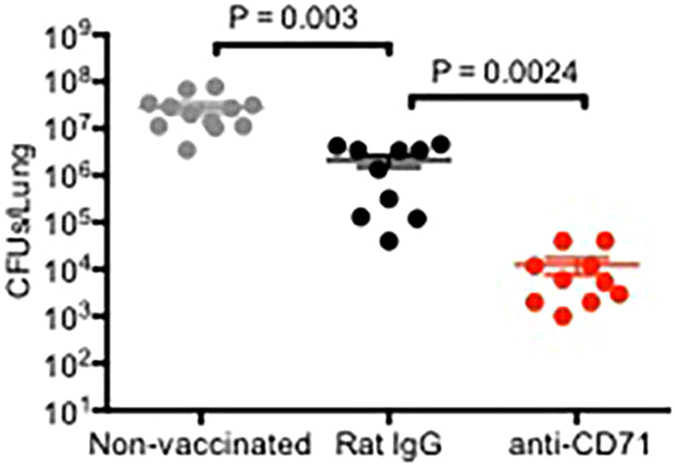
Depletion of CD71^+^ cells prior to immunization provides protection against infection with *B. pertussis*. Recoverable bacteria in the lungs of mice after infection with *B. pertussis* (5 × 10^6^ CFUs) in non-vaccinated control versus CD71^+^ cells depleted or isotype treated mice after vaccination. Each point represents data from an individual mouse, representative of three independent experiments. Bar, mean ± one standard error.

Newborn mice were either treated with anti-CD71 or Rat IgG at age of 5 days. They were then vaccinated 2 days later, boosted at age 21 and infected intranasally at age 28 days with 5 × 10^6^ CFU of live bacteria. Our data indicate that immunization in the presence of CD71^+^ cells provides minimal levels of protection in mice compared with non-vaccinated group (~1 log reduction in bacterial load) (Fig. 6), however depletion of CD71^+^ cells prior to vaccination significantly reduced bacterial burden in the lungs of mice > than 3 logs (P = 0.0024, Fig. 6).

### CD71^+^ cells suppress the functionality of T cells in human cord blood

**Figure 7:**
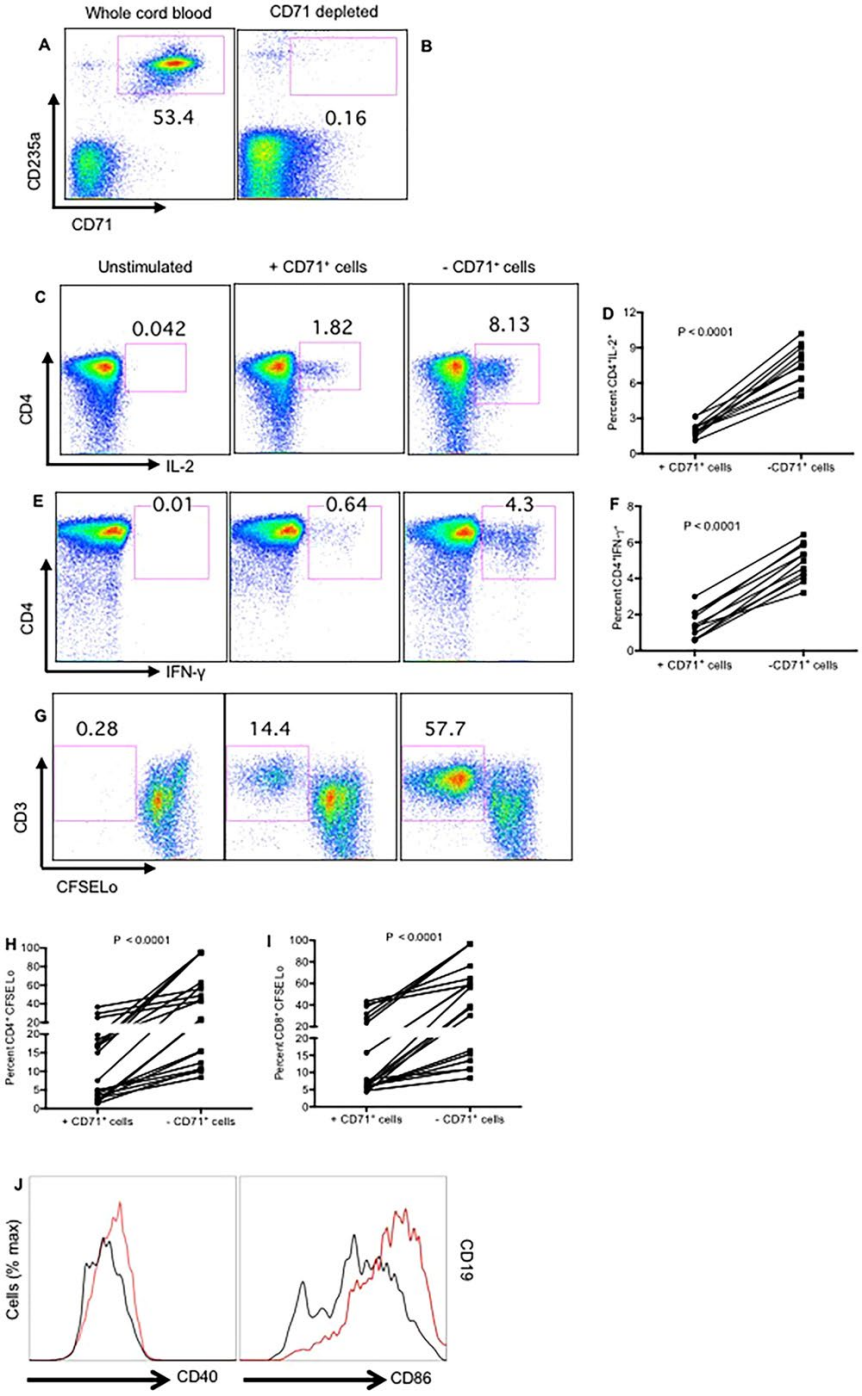
Impaired functionality of T cells and B cells by cord blood CD71^+^ cells. (**A**) Representative plots showing presence of CD71^+^CD235a^+^ cells in cord blood. (**B**) Representative plots showing depletion of these cells by cell separation techniques. (**C**) Representative plots showing IL-2 production by cord blood CD4^+^ T cells in the presence and absence of CD71^+^ cells. (**D**) Percent IL-2 producing CD4^+^ T cells from 12 individual unfractionated and CD71^+^ cell depleted cord blood specimens. (**E**) Representative plots showing IFN-γ production by cord blood CD4^+^ T cells in the presence and absence of CD71^+^ cells. (**F**) Percent IFN-γ producing CD4^+^ T cells from 12 individual unfractionated and CD71^+^ cell depleted cord blood specimens. (**G**) Representative dot plots showing percentages of CFSELo CD3^+^ in the presence and absence of CD71^+^ cells. (**H**,**I**) Percent proliferation of CD4^+^ T cells or CD8^+^ T cells respectively measured by CFSE dilution assay in the presence and absence of CD71^+^ Cells, data are from 20 cords blood specimens. Each point represents data from an individual human subject (cord blood), obtained from multiple independent experiments. (**J**) Relative CD40 and CD86 expression by cord blood CD19 cells (red histograms gated on CD19^+^ cells in the absence of CD71^+^ cells) or (black histograms gated on CD19^+^ cells in the presence of CD71^+^ cells).

Human cord blood was used to mimic the relevance of animal studies with human newborns. In consistent with our previous report^12^, human cord blood is physiologically enriched with +CD71^+^CD235a^+^ erythroid cells (Fig. 7A). CD71^+^ cells were depleted from cord blood using separation column (Fig. 7B), then the whole cord blood cells or CD71^+^ depleted cord blood cells were stimulated with SEB for 6 hours and subjected to ICS staining. Consistent with diminished responsiveness for mice immune cells in the presence of CD71^+^ cells, IL-2 (Fig. 7C,D) and IFN-γ (Fig. 7E,F) production by cord blood CD4^+^ T cells were significantly (P < 0.0001) abrogated in the presence of CD71^+^ cells. In addition, we observed that cytokine production capability of CD8^+^ T cells was suppressed by CD71^+^ cells *in vitro* (data not shown). Similar defects were found in proliferative capacity of T cells when stimulated with anti-CD3/CD28 for 4 days in the presence or absence of CD71^+^ cells (Fig. 7G–I). Finally, we found that CD19^+^ cells upregulate CD40 and CD86 in the absence of CD71^+^ cells once stimulated with HKBP for 24 h (Fig. 7J).

## Discussion

Infants are the main target age group for immunization against vaccine preventable diseases. However, they do not respond to vaccines as adults. The specific limitations of the neonatal immune responses such as blunted inflammatory cytokine production, skewed T helper differentiation, or delayed dendritic cells (DC) maturation have been associated with their impaired immune response^5^. However, it is unclear whether the diminished responsiveness to mount pathogen-specific T cell and B cell responses is due to an inherent immune cell-intrinsic defects of effector cells and APCs, or because the function of these cells is actively suppressed by suppressor cells that are present during gestation or immediately after parturition. Therefore, it is crucial to better understand about the intrinsic and extrinsic factors influencing development of adaptive immune responses in order to protect these most vulnerable populations against infectious diseases. As a proof of concept, using 6 day old newborn mice, we recently showed that neonatal CD71^+^ cells are able to abrogate innate immune responses against *B. pertussis* infection (accepted article in The J﻿ournal of Immunology). In this report, we have reconfirmed our previous finding that neonatal CD71^+^ cells express Arginase 2 and this enzymatic activity is essential for their immune-suppressive function (accepted article in the Journal of Im﻿munology). In current study, we report that CD71^+^ cells in addition to innate immunity also impair adaptive immune responses against *B. pertussis*. We found that ablation of CD71^+^ cells unleashes the expression of chemokine receptors (CXCL-1 and CXCl-2) and subsequently recruitment of APCs and CD4^+^ T cells into the lungs of newborn mice following low dose respiratory challenge with *B. pertussis*. Following re-infection with high dose *B. pertussis*, we observed greater than 2-fold increase in IL-17 production by the lung cells obtained from anti-CD71 treated mice when re-stimulated with HKBP *in vitro*. We also observed a significant threefold increase in IL-17 production by splenocytes when re-stimulated with HKBP compared to Rat IgG recipients. Similarly, when the lung cells or splenocytes were stimulated with HKBP, we detected strong induction of IFN-γ production by both the lung cells and splenocytes obtained from anti-CD71 treated versus control group. These observations were reconfirmed when CD71^+^ cells depleted newborn mice exhibited a significantly higher immune response characterized by induction of IFN-γ and IL-17 following vaccination. Thus, ablation of CD71^+^ cells resulted in a rapid and significantly greater antigen specific cytokine response following vaccination and/or re-infection with *B. pertussis*. In contrast, control mice exhibited significantly a weaker memory response to *B. pertussis* as lower IFN-γ and IL-17 secreting T cells were induced following either vaccination or re-infection. As a result, CD71^+^ cells depleted mice had significantly lower bacterial burden in their lungs compared to control group. These findings are in line with previous studies indicating that IFN-γ and IL-17 cytokines are necessary for effective clearance of *B. pertussis* ^31, 36, 37^. *B. pertussis* infection is more severe in IFN-γ^−/−^ mice^31^, as *B. pertussis* disseminates from the lungs and causes organ failure in these mice^32^. Th17 cells have been reported to play an important role in the immune response to infection with mucosal pathogens via the recruitment of monocytes and neutrophils^38^. Several recent studies have suggested that Th17 memory responses to pertussis antigens are protective^39, 40^. For example, when IL-17 mice were challenged with *B. pertussis*, the bacterial load in their lungs was 10–100-fold greater than the wild type mice^38^. We also observed that a portion of IFN-γ producing memory cells are actually IFN-γ/Th-17 co-expressing CD4^+^ T cells. This is in consistent with other recent studies showing *staphylococcus aureus*-specific memory and *Candida albicans* memory Th17 cells co-expressing IFN-γ/IL-17^41, 42^. Although, the majority of IFN-γ was secreted by memory CD4^+^ T cells, we also detected a relatively small population of CD8^+^ T cells responded to *B. pertussis* antigens which is in agreement with nonhuman primate *B. pertussis* studies^40^. However, the main question is how depletion of CD71^+^ cells enhances memory response to bacterial antigens two weeks later? Based on our data we suggest that enhanced recruitment and/or activation of innate immune cells into the lungs of anti-CD71 treated mice more likely facilitate antigen processing and presentation to T cells which improves better memory response. As we have previously demonstrated, CD71^+^ cells have a broad spectrum of immunosuppressive capabilities^12^, therefore immune cells become unleased once suppressor cells are removed/reduced. For instance, it has been reported that newborns express lower levels of surface stimulatory molecules such as CD80, CD86 and HLA-DR on their DCs^43^ which may result in impaired innate signaling such as IL-12p70 production^44^. In agreement, we have shown lower expression of stimulatory molecules on APCs and lower IL-12 production in the presence of CD71^+^ cells. However, when CD71^+^ cells are depleted, significantly higher levels of CD86, CD80 and CD40 are expressed on CD11b^+^, CD11c^+^ and B220 cells obtained from the lungs of newborn mice and subsequently more IL-12 is detected in the lung homogenates after low dose challenge with *B. pertussis*. Thus, presence of these immunosuppressor cells may explain why priming of Th1 and CD8^+^ T cell responses is diminished in newborns compared with adults^7^. These dynamic cellular encounters depend on the ability of these cells to actively migrate to the lung and it appears depletion of CD71^+^ cells facilitates recruitment and cellular interactions. On the other hand, the role of *B. pertussis*-specific antibodies in protection against primary infection is not clear and some studies suggest no protective role for these antibodies^37^. In contrast, there are other reports that demonstrated a protective role from passive and active vaccination studies in animal models^45^. Further evidence of a role for antibody against *B. pertussis* was provided when Ig^−/−^ mice failed to clear *B. pertussis* and scum to infection^45^. Interestingly, we observed detectable levels of *B. pertussis*-specific IgG and IgA antibodies in the serum and lung homogenate of mice either post re-infection or vaccination. However, only IgG levels were significantly higher in CD71^+^ cells depleted groups compared to controls in both studies. On note, we detected significantly higher levels of IgA in the serum of CD71^+^ cells depleted and vaccinated group but not in the serum of CD71^+^ cells depleted and re-infected group. We suggest this difference in IgA response might be related to the vaccine adjuvants. Although understanding the molecular mechanism of this differential effects merits further investigation, we believe lower levels of IgG antibody response to infection and vaccine might be due to immunosuppressive nature of CD71^+^ cells on B cells in control animals. For instance, lower expression of B cell stimulatory markers (e.g. CD40, CD80 and CD86) has been linked to low levels of primary IgG responses to vaccines and/or infections^46^. In agreement, we found depletion of CD71^+^ cells unleashed expression of CD40, CD80 and CD86 on neonatal B cells.

Because of the immunosuppressive nature of CD71^+^ cells, we next investigated whether CD71^+^ cells in human cord blood also exhibit suppressive functions against T and B cells *in vitro*. In consistent with animal studies, we found that removal of cord blood CD71^+^ cells unleashes cytokine production ability and proliferative capability of both CD4^+^ and CD8^+^ T cells following stimulation *in vitro*. We also detected upregulation of costimulatory molecules (e.g. CD40, CD80 and CD86) following stimulation of B cells with Toll like receptor ligands such as HKBP in the absence of CD71^+^ cells. This suggest that the immune suppression mediated by CD71^+^ cells may prevent activation of B cells via T cell-dependent or T cell-independent pathways.

The present findings, in agreement with previous reports, suggest that the immune response to natural pertussis infection or vaccination is skewed towards Th17 and Th1 and *B. pertussis*-specific antibody responses. Importantly, this is the first study to show that CD71^+^ cells suppress adaptive immunity against *B. pertussis*. Our novel report shed important light on the role of CD71^+^ cells in adaptive immunity. Our findings may have implications for further studies in providing the newborn with optimal protection against infectious diseases.

## Methods

### Animals

Male and female BALB/c mice, were purchased from the Charles River Laboratories (St. Constant, QC, Canada) for this study. All mice were housed in specific pathogen-free environment within the animal care facility at the University of Alberta. BALB/c mice were bred together, and pregnant mice were checked daily to establish birth timing. At five days of age, newborn mice were transferred to a biohazard facility and housed in cages during the vaccination and challenge with *B. pertussis*. This study was carried out in strict accordance with the recommendations in the Guide for the Care and Use of Laboratory Animals of the Canadian Council for Animal Care. Animal protocol was approved by the University of Alberta Animal Policy and Welfare Committee in accordance with the Canadian Council on Animal Care guidelines (Protocol #AUP00001021).

### Cord blood

Cord blood was obtained from full-term deliveries for these studies. The appropriate Institutional Review Boards at the University of Alberta approved the studies. All study participants gave written informed consent to participate in this study.

### Bacterial Culture

*B. pertussis* strain Tohama I was grown on Charcoal agar (Oxoid Inc., ON, Canada) containing 10% defibrinated sheep blood. Bacterial cultures were incubated under aerobic conditions at 37 °C for 48 h followed by a subculture of bacterial scrapes in Stainer-Scholts (SS) media overnight. Bacterial suspensions were prepared for infection as described previously^13, 14, 47^, and kept on ice prior to the challenge. Heat-killed *B. pertussis* (HKBP) suspensions were also prepared for vaccination, cell stimulation and coating ELISA plates. For this purpose, *B. pertussis* was grown in SS media to early log phase, harvested, washed and resuspended in sterile saline. Then, the bacteria were killed by incubation at 56 °C for 30 minutes. Each batch was verified to be sterile by plating onto Charcoal agar, and stored at −20 °C before use.

### Preparation of experimental vaccine

The triple combo vaccine with previously demonstrated protective immunity against *B. pertussis* was used in this study^34, 35^. This vaccine consisted of pertussis detoxified toxin (PTd) was kindly provided by Novartis vaccines (Sienna, Italy), HKBP, poly I:C (polyinosinic:polycytidylic acid, InvivoGen), cationic peptide IDR-1002 (VQRWLIVWRIRK-NH2, GENSCRIPT, USA Inc. (Picataway, NJ, USA)). Newborn mice (7 day old) were subcutaneously (s.c.) immunized between the shoulder blades and boosted two weeks later in the similar manner.

### Anti-CD71 treatment and respiratory infection

Five day old pups were injected intraperitoneally with either 200 µg purified anti-CD71 (8D3) or Rat IgG2a isotype control antibodies for the *in vivo* depletion of CD71^+^ cells. Two days after the anti-CD71 treatment (day 7 after birth), randomly selected newborns were euthanized, and the depletion of CD71^+^ cells was confirmed in spleen and lungs. The rest of the anti-CD71 treated and IgG2a control newborns were infected with *B. pertussis* intranasally by carefully placing 25 µl of ~5 × 10^2^ CFUs bacterial suspension on the top of each nostril, and allowed to be inhaled. A second infection was applied at day 21 after birth, however, a higher dose of the bacterial suspension (25 µl of 5 × 10^6^ CFUs) was administered. All mice were euthanized at day 4 post re-infection, lung and spleen samples were harvested for further analyses.

### Lung and spleen samples processing

For the enumeration of bacterial recovery in the infected mice, lungs were thoroughly disrupted with tissue homogenizer in 2 ml of SS media. Then, the suspension was used to generate 10-fold serial dilutions in duplicates onto Charcoal agar and incubated at 37 °C for 4 to 5 days. For the quantification of cytokines and flow cytometric analysis, lung samples were homogenized in 2 ml of PBS containing protease inhibitor. Subsequently, the lung homogenates were centrifuged at 1200 × g for 10 min and cell pellets were collected for flow cytometric analysis, whereas supernatants were stored at −20 °C for the determination of cytokine and antibody levels. To obtain single-cell suspensions, spleen samples were grinded between sterile frosted glass slides in 7 ml of 1x RBC lysis buffer, and then filtered through nylon mesh.

### Antibodies and flow cytometry

Fluorophore conjugated antibodies with specificity to mouse and human cell surface antigens and cytokines were purchased from eBioscience or BD Biosciences. For surface staining, isolated cells from spleen and lungs were stained with anti-CD3 (SK7), anti-CD4 (GK1.5), anti-CD8a (53–6.7), anti-CD11b (M1/70), anti-CD11c (N418), anti-CD71 (R17217 and C2F2) and anti-TER119 (TER-119) in PBS with 2% fetal bovine serum (FBS) (Sigma-Aldrich). For intracellular staining, splenocytes and lung cells were initially stained with surface markers, and then fixed and permeabilized with Cytofix/Cytoperm buffer (BD Bioscience). Prior to this step, cells were cultured at 1 × 10^6^ cells ml^−1^ in DMEM media supplemented with 10% FBS (Sigma-Aldrich) and 10 µg ml^−1^ brefeldin A. Then, splenocytes suspensions were stimulated or not with 10 µl of HKBP and incubated for 5 h at 37 °C. The intracellular staining was performed using anti-IFN-γ (XMG1.2), anti-IL-17a (eBio17B7) and anti-IL-2 (MQ1–17H12). At the last step of each staining type, cells were fixed with paraformaldehyde, acquired by flow cytometry using a LSRFORTESSA flow cytometer (BD Biosciences) and analyzed with FlowJo software.

### Determination of IgG and IgA antibodies specific to *B. pertussis* toxin

IgG and IgA antibodies specific to *B. pertussis* toxin were quantified in serum and lung homogenates by ELISA. In brief, a suspension of HKBP was sonicated 7 times for 30 s and centrifuged at 1200 rpm for 10 min. The collected supernatant was used to measure the *B. pertussis* toxin by BCA protein assay (Thermo Fisher Scientific). Then, 96-well plates were pre-coated with 100 µl of the HKBP toxin at a concentration of 4 µg/ml in sodium carbonate coating buffer (pH = 9.6) and incubated overnight. After washing, the plates were blocked for 60 min with PBS with 10% bovine serum albumin (Sigma-Aldrich), followed by another washing 3 times and incubation of the serum samples and lung homogenates for 2 h at room temperature. The detection of the immobilized *B. pertussis* toxin-specific antibodies was performed by incubating the plates with biotin labelled anti-mouse IgG or IgA conjugates (Mabtech) for 1 h and 30 min. Streptavidin-HRP (Mabtech) was used as a secondary antibody and incubated for 20 min at the dark followed by another washing. Then, a substrate (R&D Systems) was added and catalyzed by the HRP to generate the enzymatic signal, and 2 N H_2_SO_4_ was used to stop the reaction. The optical density of each well was determined immediately using a microplate reader set to 450 nm.

### *In vitro* cytokine measurements

Cytokine responses were measured by performing ELISpot or intracellular cytokine secretion (ICS) assays and IFN-γ and IL-17 ELISpot assays were used for the screening of an immune responses in the vaccine treated, *B. pertussis* infected and control animals. Both splenocytes and lung cells were cultured at the concentration 1 × 10^5^ cells per well and stimulated with 2 µg ml^−1^ HKBP overnight. ELISpot kit (ImmunoSpot) was employed to measure the two cytokines as we previously described^48^, and the analysis of the plates was performed using CTL ImmunoSpot Analyzer (Cellular Technology Ltd Supplier). Positive responses were designated when the number of spot-forming cells was twice the background and at least 50 spot-forming cells/10^6^. In brief for ICS, 1 × 10^6^ cells per well were cultured and stimulated with 2 µg/ml of HKBP for mice cells or 100 ng/ml of Staphylococcal Enterotoxin B (SEB) for human cells.

### Proliferation assays

Cord blood samples were collected under sterile conditions and CBMC were separated from whole-blood by density centrifugation on Ficoll-Paque. The isolated mononuclear cells were used for positive CD71 enrichment with biotin labelled CD71 antibody (OKT9) and LS separation columns (Miltenyi Biotech). Whole-blood, CD71 enriched, and CD71 negative cells were labeled with CFSE, plated in RPMI 1640 supplemented with 10% FBS, 100 U per ml penicillin-streptomycin solution (Sigma-Aldrich) at concentration 2 × 10^6^ cells per well, and stimulated with anti-CD3 (Clone UCHT1) and anti-CD28 (Clone CD28.2) antibodies for 4 days. Then, proliferated T cells were analyzed as we previously described^48, 49^.

### Tissue RNA extraction, cDNA synthesis and quantitative PCR

Lung tissues were homogenized with 2.8 mm stainless steel beads using a FastPrep-24 (MP Biomedicals, Solon, OH) in 3 cycles of 30-second bead-beating step at 4 m/s speed. RNA from lung homogenates was extracted in TRIzol (Sigma) using the RNeasy Mini Kit (Qiagen, Courtaboeuf, France), and 1 ug RNA was converted to cDNA by reverse-transcription using QuantiTect Reverse Transcription kit (Qiagen). cDNA was then subjected to quantitative PCR using TaqMan Fast Advanced Master Mix (Applied Biosystems-Foster City, CA, USA) with TaqMan probes for CXCL-1 (Mm04207460_m1), CXCL-2 (Mm00436450_m1), CCL-2 (Mm99999056_m1), CCR-7 (Mm0043608_m1), and TLR-4 (Mm00445273_m1). Samples were analyzed in duplicates and run on CFX96 TouchTM Real-Time PCR Detection System (BioRad). The results were calculated as a fold change in gene expression relative to uninfected condition using the 2^−ΔΔCt^ method, where glyceraldehyde phosphate dehydrogenase (GAPDH) was used as a reference gene^50^.

### Statistical analysis

Graphical presentation and statistical analysis of data was performed using GraphPad Prism version 6.00. To detect differences between treated and control groups, Student’s *t* test was applied. One-way ANOVA followed by Tukey’s test for multiple comparisons was performed to check for significant differences when more than two groups were compared. Results were expressed as means with their standard errors. A *p* value of 0.05 was considered statistically significant.
